# Experimental validation of acoustic and thermal modeling in heterogeneous phantoms using the hybrid angular spectrum method

**DOI:** 10.1080/02656736.2021.2000046

**Published:** 2021

**Authors:** Megan Hansen, Douglas Christensen, Allison Payne

**Affiliations:** a Department of Radiology and Imaging Sciences, University of Utah, Salt Lake City, UT, USA;; b Department of Biomedical Engineering, University of Utah, Salt Lake City, UT, USA;; c Department of Electrical and Computer Engineering, University of Utah, Salt Lake City, UT, USA

**Keywords:** Acoustic modeling, high-intensity focused ultrasound, tissue-mimicking phantoms, acoustic properties

## Abstract

**Purpose:**

The aim was to quantitatively validate the hybrid angular spectrum (HAS) algorithm, a rapid wave propagation technique for heterogeneous media, with both pressure and temperature measurements.

**Methods:**

Heterogeneous tissue-mimicking phantoms were used to evaluate the accuracy of the HAS acoustic modeling algorithm in predicting pressure and thermal patterns. Acoustic properties of the phantom components were measured by a through-transmission technique while thermal properties were measured with a commercial probe. Numerical models of each heterogeneous phantom were segmented from 3D MR images. Cylindrical phantoms 30-mm thick were placed in the pre-focal field of a focused ultrasound beam and 2D pressure measurements obtained with a scanning hydrophone. Peak pressure, full width at half maximum, and normalized root mean squared difference (RMSDn) between the measured and simulated patterns were compared. MR-guided sonications were performed on 150-mm phantoms to obtain MR temperature measurements. Using HAS-predicted power density patterns, temperature simulations were performed. Experimental and simulated temperature patterns were directly compared using peak and mean temperature plots, RMSDn metrics, and accuracy of heating localization.

**Results:**

The average difference between simulated and hydrophone-measured peak pressures was 9.0% with an RMSDn of 11.4%. Comparison of the experimental MRI-derived and simulated temperature patterns showed RMSDn values of 10.2% and 11.1% and distance differences between the centers of thermal mass of 2.0 and 2.2 mm.

**Conclusions:**

These results show that the computationally rapid hybrid angular spectrum method can predict pressure and temperature patterns in heterogeneous models, including uncertainties in property values and other parameters, to within approximately 10%.

## 1. Introduction

The use of magnetic resonance-guided focused ultrasound (MRgFUS) to treat various pathologies is increasing rapidly [1], with current clinical treatment of Parkinsonian and essential tremors [2,3], uterine fibroids [4], prostate tissue [5,6], bone metastases [7], breast diseases [8,9], and desmoid tumors [10]. Accurate targeting of the focused ultrasound beam is critical to ensure treatment safety and efficacy. The complexity and accuracy of treatment planning, including both acoustic and thermal modeling, varies between equipment vendors and anatomical sites. Increased accuracy and utility of treatment planning can be achieved with acoustic and thermal modeling techniques that incorporate patient-specific anatomy and properties [11,12]. While there are many established accurate acoustic modeling techniques, their usefulness in clinical treatment planning and analysis depends to a large extent on their computational efficiency and speed.

For accurately modeling pressure waves in homogeneous tissues, there are numerous beam propagation techniques described in the literature. For instance, the Rayleigh-Sommerfeld integral [13] is a well-established method that performs linear wave propagation in homogeneous media. Current implementation of the Rayleigh-Sommerfeld integral with GPU-based computing libraries has substantially reduced computational times [14]. In [14], a multi-layer approach of the Rayleigh integral combined with a finite-difference time-domain implementation of the Pennes bioheat transfer equation allowed for reasonably timed MRgFUS treatment planning activities. For more complex tissue constructs that include voxel-wise inhomogenieties and non-linearities, several beam propagation equations may be applied, including the Khokhlov-Zabolotskaya-Kuznetsov (KZK) [15,16] and Westervelt [17,18] formulations. Simulation methods to account for inhomogeneities and non-linear responses include finite-difference time-domain (FDTD) [19], the Wen-Breazeale method of Gaussian decomposition [20] and the pseudospectral k-Wave technique [21]. There have been significant advances in reducing computational time for many of these techniques using the parallelization advances of graphics processing units.

The traditional angular spectrum approach performs beam propagation calculations in the spatial frequency domain [22], and while the inherent computational efficiency of fast Fourier transforms makes this method both rapid and accurate, the traditional angular spectrum technique cannot be directly applied to heterogeneous tissue models, limiting its application in patient-specific modeling. The hybrid angular spectrum (HAS) method [23] is a beam modeling technique that extends the traditional angular spectrum method to heterogeneous applications by modeling the heterogeneous media as small voxels, each with unique acoustic properties of speed of sound, attenuation, and density. Wave propagation is performed in a split-step manner, progressing along the beam propagation direction through successive transverse planes. For each plane of voxels, the propagation is calculated by accounting for the components of the phase shift in two different steps: one in the space domain using voxel-specific phase variations from the average phase shift over the plane (also including voxel-wise attenuation effects) and one in the spatial frequency domain using the planar-averaged phase to propagate to the next plane. HAS makes several key assumptions to achieve computational efficiency, including linear propagation, property uniformity within a voxel, property uniformity over time, steady-state conditions, and propagating waves that do not spread out into large, divergent angles.

The HAS technique has been computationally compared to acoustic FDTD simulations, demonstrating a normalized root mean square difference of 2.8% over a 301 × 301 × 300-voxel 3D breast model [23]. Additionally, HAS-simulated pressures have been compared to experimentally obtained pressures for propagation through an *ex vivo* skull [24], a photopolymer aberrator [25] and homogeneous gelatin phantoms [26]. However, these studies presented beam patterns whose magnitudes were self-referenced to the maximum value of each pattern instead of absolute measurement comparisons. Experimental temperature patterns have been compared to HAS results for homogeneous gelatin [26] and heterogeneous gelatin breast-shaped phantoms with canola oil inclusions [25] as well as a retrospective study comparing temperatures of transcranial ultrasound procedures [27]. In the heterogeneous cases [25,27], it was found that a subject-specific multiplicative scale factor was necessary to reasonably correlate the experimental to simulated temperature results.

This study aims to quantify the accuracy of focused ultrasound treatment planning using the hybrid angular spectrum method in controlled homogeneous and heterogeneous phantom environments by directly comparing simulations with experimental pressure and temperature patterns. In the many practical applications where inexactness due to the approximations of the HAS method is less or no greater than other uncertainties in the simulation, the computational efficiency of the HAS algorithm provides potential clinical utility in patient-specific treatment planning performed during focused ultrasound treatments.

## 2. Materials and methods

Homogeneous and heterogeneous tissue-mimicking phantoms were constructed in three different sizes to accommodate the different measurement and testing techniques required to model and assess both the pressure predictions using the HAS simulation technique and the resulting thermal response during MRgFUS sonications.

### 2.1. Phantom design and construction

The phantoms were constructed based on a previously characterized gelatin recipe designed to model human tissue [28]. As detailed in Table 1, three phantom types, Witness (W-Type), Pressure (P-Type), and Thermal (T-Type), were used to accommodate the different testing conditions. Molds were constructed of acrylic cylinders whose ends were covered with clear vinyl or mylar film (0.085-mm and 0.1-mm thickness, respectively) and sealed with silicone adhesive. All phantoms were fabricated from porcine gelatin (250-bloom ballistics gelatin, Vyse Gelatin Co., Schiller Park, IL). The homogeneous phantoms were made using three evaporated milk concentrations (Nestlé Carnation Evaporated Milk, 30%, 50%, and 70% by volume) to mimic different acoustic attenuation values (*N* = 9 total, *N =* 3 at each milk concentration). For each mixture, a 500-ml gelatin batch was divided into a W-Type phantom for speed of sound, attenuation and thermal property characterization, and a P-Type phantom for pressure measurements by hydrophone. The gelatin was poured through side access holes and cooled.

**Figure 1.**
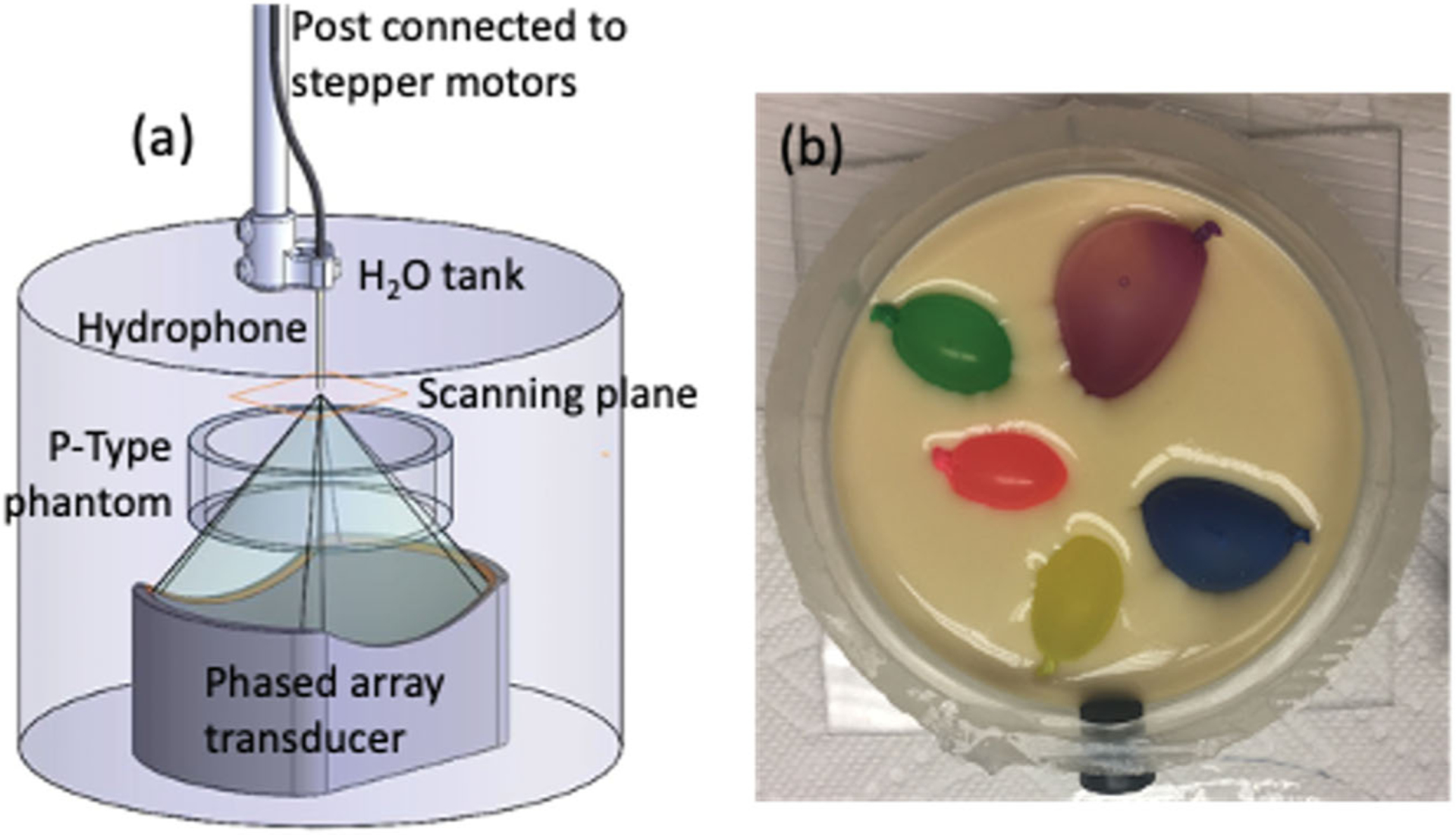
Schematic of the experimental setup used for hydrophone scanning. (a) Hydrophone scanning setup with the placement of the thinner P-Type phantom shown in the testing column. (b) Photo of one heterogeneous P-Type phantom with exposed canola-oil inclusions before the final gelatin pour. Phantom diameter is 10.2 cm with a height of 3 cm. The testing column suspended the phantom such that positional accuracy was assured under all testing conditions.

The heterogeneous phantoms were made in both P-Type (*N* = 3) and T-Type (*N =* 2) configurations. They contained gelatin with 70% milk by volume along with several variously sized canola-oil-in-balloon inclusions, as previously described [25]. The 70% milk mixture was used exclusively in the heterogeneous phantoms to emphasize the acoustic contrast with the canola-oil balloons. A photo of a P-Type heterogeneous phantom in mid-construction is shown in Figure 1(b). During fabrication, the gelatin was poured in layers and cooled, allowing the canola-oil inclusions to be placed between layers before the phantom was topped-off and sealed.

### 2.2. Material property determination

Acoustic and thermal property characterization was performed on each gelatin and canola-oil component of the phantoms using techniques that have been previously described [25]. In particular, through-transmission testing was employed to measure the speed of sound and attenuation of the materials using the W-Type phantoms [28]. The density was calculated by dividing phantom mass by calculated cylinder volume. After through-transmission testing with the W-Type phantoms, a commercially available device (KD2 Pro Thermal Properties Analyzer, Decagon Devices, Pullman, WA) pierced the ends of the phantoms for transient-line-source measurements of thermal diffusivity. This device has two 30-mm-long probes spaced 6 mm apart, with one probe being used for heating and the other for temperature measurements. Thermal measurements were not possible in the canola oil with this tool and therefore previously published values were used [29]. The measurement uncertainty of each technique was determined based on the variability of the experimental data or the manufacturer’s reported precision.

### 2.3. Pressure measurements

A scanning hydrophone (HNR-0500, Onda Corporation, Sunnyvale, CA) was used to directly measure the 2D pressure patterns of the ultrasound beam after propagation through the P-type phantoms. Both the homogeneous and heterogeneous P-Type phantoms were placed in the pre-focal zone of a focused ultrasound transducer mounted vertically in a degassed water-filled testing column, as seen in Figure 1(a). Phantoms were supported by a platform designed to avoid interference with the propagating beam and to secure the phantom’s position, ensuring positional consistency across each testing configuration. The focused ultrasound transducer was a 256-element phased array (Imasonic, Voray-surl’Ognon, France; frequency: 940 kHz; focal length: 10 cm; aperture: 14.4 × 9.8 cm; full-width-half-maximum intensity pressure pattern: 1.8 × 2.5 × 10.9 mm in water; acoustic power: 2.3 W) [30]. The deionized water in the testing column was degassed prior to testing (<2 ppm) to prevent beam scattering and hydrophone damage. A 2D scan with 0.25-mm isotropic resolution over a 1.0 × 1.0-cm area was centered around the focal point of the beam. The measured 2D complex pressure pattern was then propagated into a 3D volume matching the relevant portion of the simulated model size using the angular spectrum approach [22] for comparison with HAS- and k-Wave-simulated patterns.

### 2.4. MRgFUS sonications and MR thermometry

MRgFUS heating sonications were performed using the T-Type phantoms inside a Siemens 3 T Prisma^FIT^ scanner (Siemens Healthcare, Erlangen, Germany). The phantoms (*N* = 2) were placed in the testing column seen in Figure 1(a) and suspended above the MRI-compatible 256-element phased-array transducer described above. An 8-channel flexible receive coil array was placed around the testing column to provide sufficient imaging signal-to-noise ratio. The transducer was coupled to the face of the phantom with degassed, deionized water. An MR-compatible power generator (Image Guided Therapy, Pessac, France) was used to control the MRgFUS system. For each T-Type phantom, three sonications were applied at the geometric focus of the transducer (20.84 s, 50.1 ± 0.1 acoustic W each). Approximate 5-minute cooling intervals were allowed between sonications. The thermal response of each sonication was monitored in real time using 3D MR temperature imaging (3D GRE with segmented EPI readout; TR/TE: 38/11 ms; resolution: 1.5 × 1.5 × 3 mm; FOV: 80 × 128 × 20 mm; flip angle: 15°; acquisition time: 5.5 s/volume; ETL: 7). All MR temperature imaging (MRTI) data were interpolated to 0.5-mm isotropic resolution [31] before analysis.

### 2.5. Computational model creation

**Figure 2.**
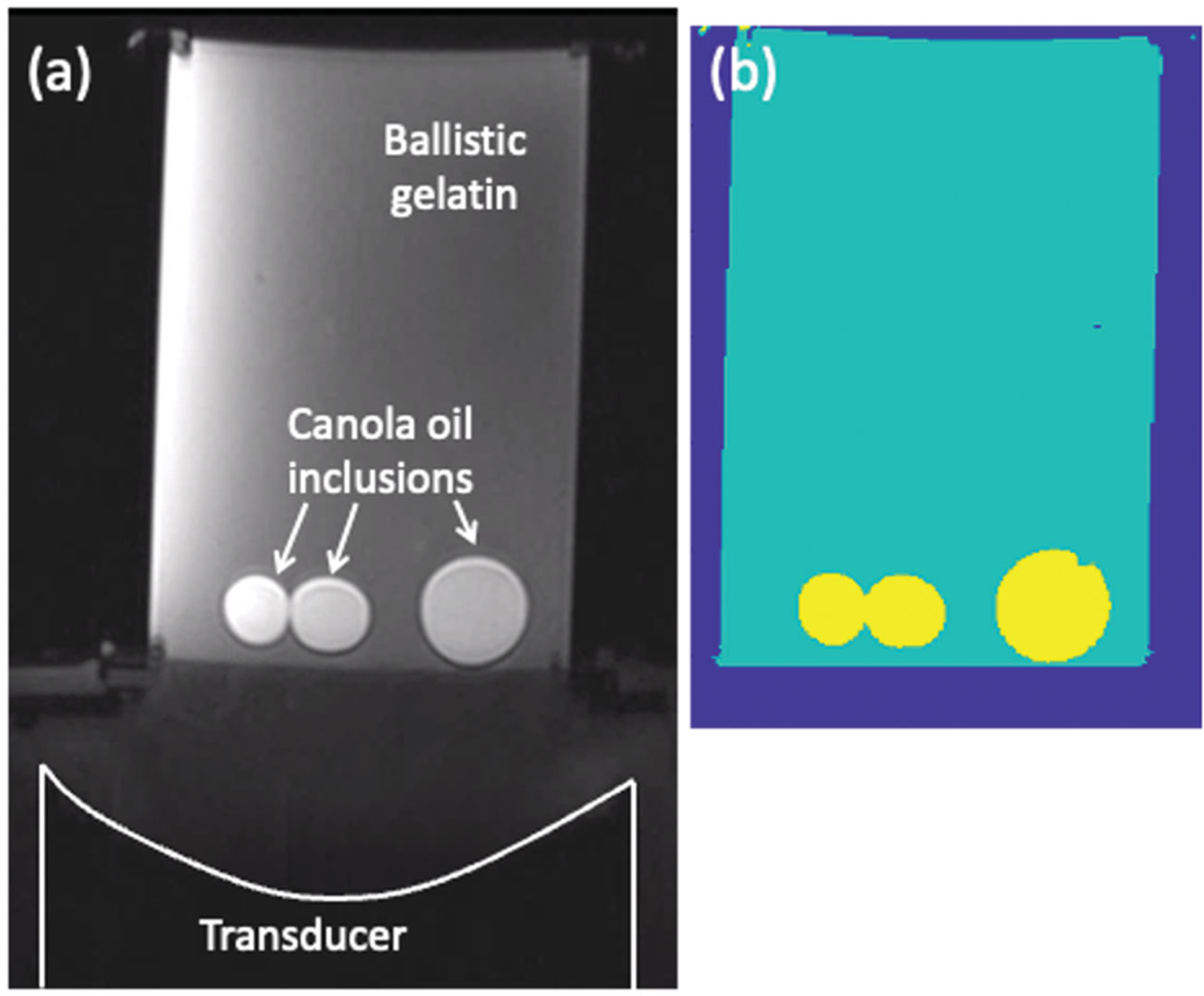
A representative T-Type heterogeneous phantom (102 × 150 mm) used for the MRgFUS sonication studies. (a) Axial T1w image of the phantom in the testing column with the focused ultrasound transducer, the ballistic gelatin and the canola-oil inclusions labeled. (b) Segmented computational model with three material types: water, gelatin and canola oil. Resolution of both images is 0.25 mm isotropic.

Segmented computational models were created for acoustic and thermal simulations for both the heterogeneous P-Type and T-Type phantoms. Because the phased-array transducer and heterogeneous phantoms were rigidly aligned together in the testing column, MR-imaging of the arrangement allowed registration of the simulation models to experimentally acquired data. Volumetric MRI scans (T1-weighted 3D VIBE 2-point Dixon; TR: 20 ms; TE: 2.46/3.69 ms; flip angle: 25°; 256 × 240 × 176 mm; 1-mm isotropic resolution; band-width: 350 Hz/pixel) were obtained of each phantom in the testing column. MRI data were interpolated to 0.25-mm isotropic resolution and the phantom was semi-automatically segmented into gelatin and canola oil materials using intensity thresholding of the fat- and water-separated images (Seg3D, version 2.4.4, NIH Center for Integrative Biomedical Computing, SCI Institute, University of Utah [32]). The transducer face was visible in the MR images, allowing for precise determination of the transducer position with respect to the phantom. For the T-Type heating phantoms, four independent observers calculated the geometric focus of the transducer using high-resolution MR images of each imaged phantom configuration. Figure 2 shows a representative MR image and the corresponding segmented model for one of the T-Type phantoms.

### 2.6. Acoustic simulations

Acoustic simulations in this study were done with the HAS algorithm [23] for all phantom configurations along with k-Wave version 1.3 [21] for the heterogeneous P-Type phantoms. The input parameters to the acoustic simulations, including the model position with respect to the transducer and the material acoustic properties, were determined by the previously described measurement and characterization techniques. The acoustic attenuation coefficient was assumed to be purely absorptive with negligible scattering. Attenuation values at 1.0 MHz were linearly scaled to apply at the 940-kHz frequency of this study. For the k-Wave simulations, the source pressure at the front face of the model was the same as used for the HAS simulations, obtained by a one-time Rayleigh-Sommerfeld integral of the transducer output field, scaled to accommodate various transducer power levels. The k-Wave simulations were allowed to go to steady state and the pattern of maximum pressure was stored during the last two or three complete cycles for comparison with the HAS results. All pressure simulations were performed at a computational resolution of 0.25 mm isotropic.

For the P-Type phantoms, both homogeneous and heterogeneous, the simulated 3D pressure patterns were directly compared to those derived from the scanning hydrophone measurements. For the T-Type phantoms, the simulated pressure patterns *p* (in units of Pa) were combined with the local acoustic impedance *Z* (in Rayl) and absorption coefficient α (in Np/m) to calculate the deposited power density *Q* (in W/m^3^) and thus the heat deposition term in the Pennes bioheat transfer equation [33] using the relation *Q* = α*p*^2^=*Z* [34], allowing thermal simulations to be performed.

### 2.7. Thermal simulations

A finite-difference time-domain implementation (0.1-s temporal resolution; 0.5-mm isotropic spatial resolution) of the Pennes bioheat transfer equation was used to simulate the MRgFUS sonication temperatures performed in the T-Type phantoms. Inputs included the power deposition pattern, adiabatic boundary conditions, a uniform initial condition, and the material-specific thermal properties directly measured from the phantoms or taken from the literature (see Table 2). The output was a 3D temperature pattern as a function of time during and immediately after the sonication period.

### 2.8. Data analysis – pressures

Comparisons were made between the beam pressure patterns obtained by simulation and hydrophone scanning after propagation through the homogeneous and heterogeneous P-Type phantoms. Peak pressure values and the full-width at half-maximum (FWHM) widths in all three directions were found and the average differences between simulated and experimental values within each phantom category (3 × 3 homogeneous phantoms and three heterogeneous phantoms) are reported as a mean ± one standard deviation. In addition, for the comparison of pressure patterns, a normalized root mean square difference (*RMSDn*) was calculated:

(1) $$\text{RMSDn}=\;\sqrt{\frac{\sum_{i=1}^{N}{\left(\frac{p_{sim,i}-p_{\text{exp},i}}{p_{max,\text{exp}}}\right)}^{2}}{N}},$$

where *p*_*sim,i*_ and *p*_*exp,i*_ are the absolute magnitudes of the simulated and experimental pressures at voxel *i*, *N* is the total number of voxels in a 41 × 41 × 41 region around the focus, and *p*_*max,exp*_ is the global maximum pressure determined experimentally. Importantly, the individual pressure patterns were not self-normalized before entry into this equation.

To further quantify the similarity between the spatial distributions of the heterogeneous pressure patterns on a particular plane, an Earth Mover’s Distance (EMD) metric was employed [35] as is sometimes used in image retrieval. This metric specifies the minimum effort, denoted as “work,” required to transform one pressure spatial distribution, or “mass” pattern, into another. In this case, “work” is measured as the total product of the normalized pressure values that need to be moved times the physical distance (the “ground distance”) they are moved to change one pattern into the other. The lower the EMD, given in units of pixel length, the closer the two patterns are to each other in their pressure distributions.

### 2.9. Data analysis – temperature

The predictive accuracy of the HAS algorithm in the heated T-Type phantoms was quantified by several metrics. The temporal responses of the simulated and experimental temperatures at the peak temperature location and averaged over a 16-mm^3^ region surrounding the peak temperature location were compared. The distance between the centers of thermal mass [25] of the experimentally measured and simulated temperatures was also calculated. Finally, the root mean square difference (RMSD) was calculated with non-normalized temperatures *t* as the variables,

(2) $$\text{RMSD}=\sqrt{\frac{\sum_{i=1}^{N}(t_{sim,i}-t_{\text{exp},i}{)}^{2}}{N}},$$

as well as the normalized root mean squared difference, similar to Equation (1). These metrics are presented for each of the three sonications followed by a mean over the sonications for each of the T-Type phantoms.

## 3. Results

The material property values for the different gelatin mixtures and the canola oil used in the phantoms are reported in Table 2 as a mean value ± one standard deviation. The thermal conductivity and specific heat values for gelatin with 30% and 50% milk are not reported since no heating simulations were performed with those materials. While the mean is reported here, phantom-specific properties were used in simulations since the gelatin mixture for each phantom was measured separately.

Table 3 reports the comparison metrics for the hydrophone-measured and simulated pressure patterns using both HAS and k-Wave after propagation through the homogeneous and heterogeneous P-Type phantoms. For the homogeneous phantoms, the average variation between the experimental and simulated pressure patterns was better than 6% for all metrics (peak pressure, FWHM, and normalized RMSD). The values obtained with these simpler uniform phantoms approximates the level of variance that can be expected taking into account such uncertainties as medium properties, hydrophone characterization, transducer power and experimental measurement.

For the heterogeneous P-Type phantoms where the (non-phase-corrected) beams are strongly aberrated, the average variation seen in Table 3 was greater, 9.0% for peak pressures and 11.4% RMSDn for HAS compared to hydrophone, but a smaller 5.0% RMSDn for HAS compared to k-Wave patterns.

**Figure 3.**
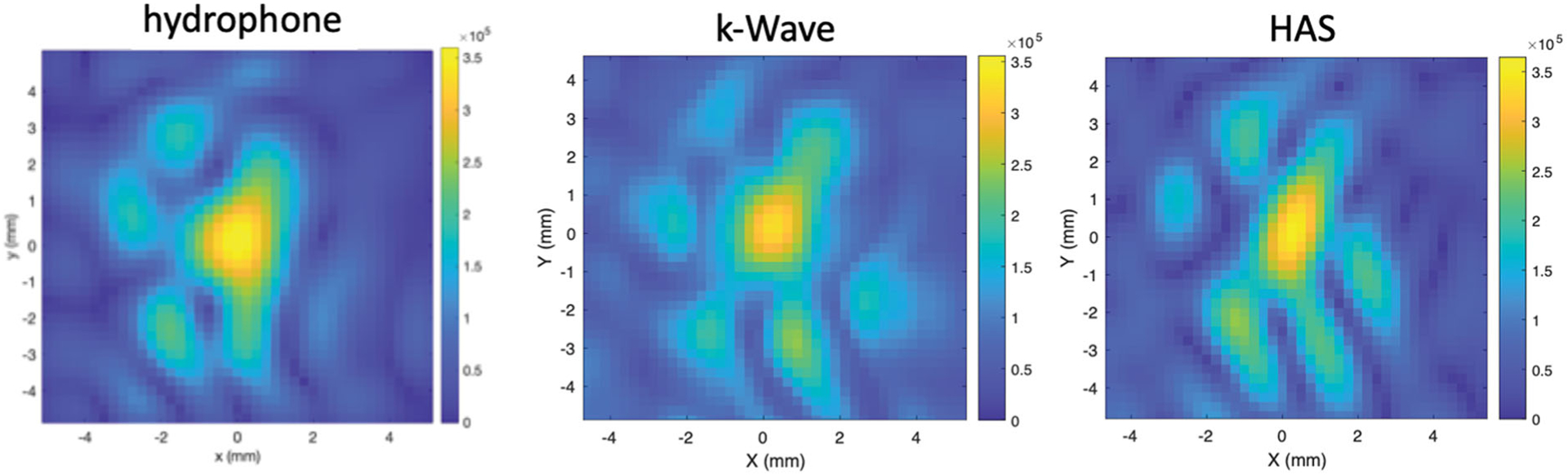
Pressure patterns (in Pa) in the transverse plane centered on the geometric focus comparing the hydrophone measurement results with k-Wave and HAS simulations of the ultrasound beam after propagation through one of the heterogeneous P-Type phantoms.

Further, to illustrate a typical example of the aberrated patterns seen around the geometric focus of the transducer, Figure 3 shows images of the absolute value of the pressure pattern on a transverse plane at the geometric focus as obtained by the HAS method, hydrophone scanning and k-Wave for one of the heterogeneous P-Type phantoms. Although significantly aberrated, all three beam patterns show clear similarities in the position and sizes of the lobes. To quantify these similarities, an Earth Mover’s Distance (EMD) analysis was performed pair-wise on these three images. The EMD between the HAS and the k-Wave patterns was 0.9, between the HAS and the hydrophone patterns was 1.3, and between the k-Wave and hydrophone patterns was 1.2, all values in units of pixel length. As mentioned earlier, the smaller the EMD value, the closer the patterns match. To give perspective to these values, the EMD calculated between the HAS pattern and the hydrophone pattern rotated 90° (representing two patterns that are not well matched) was found to be 1.8.

**Figure 4.**
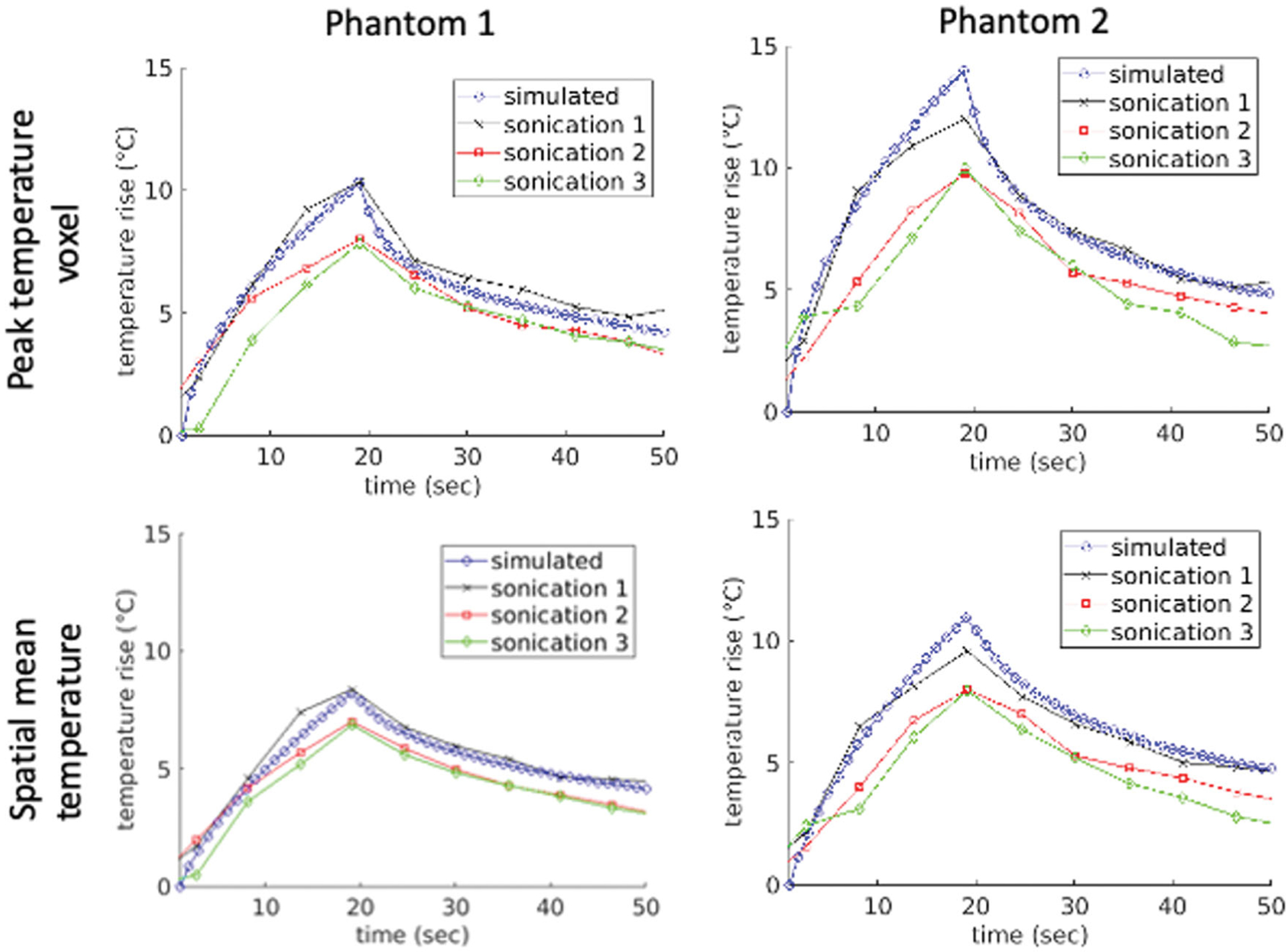
Experimental peak and mean temperatures measured with MRTI compared to simulated values during MRgFUS sonications in the heterogeneous T-Type phantoms. The upper figures plot the peak temperature voxel for each of the individual sonications along with the mean of the simulated values for Phantoms 1 and 2. The lower figures display the spatial temperature means over a 16-mm^3^ cubic volume centered at the peak temperature point for Phantoms 1 and 2.

**Figure 5.**
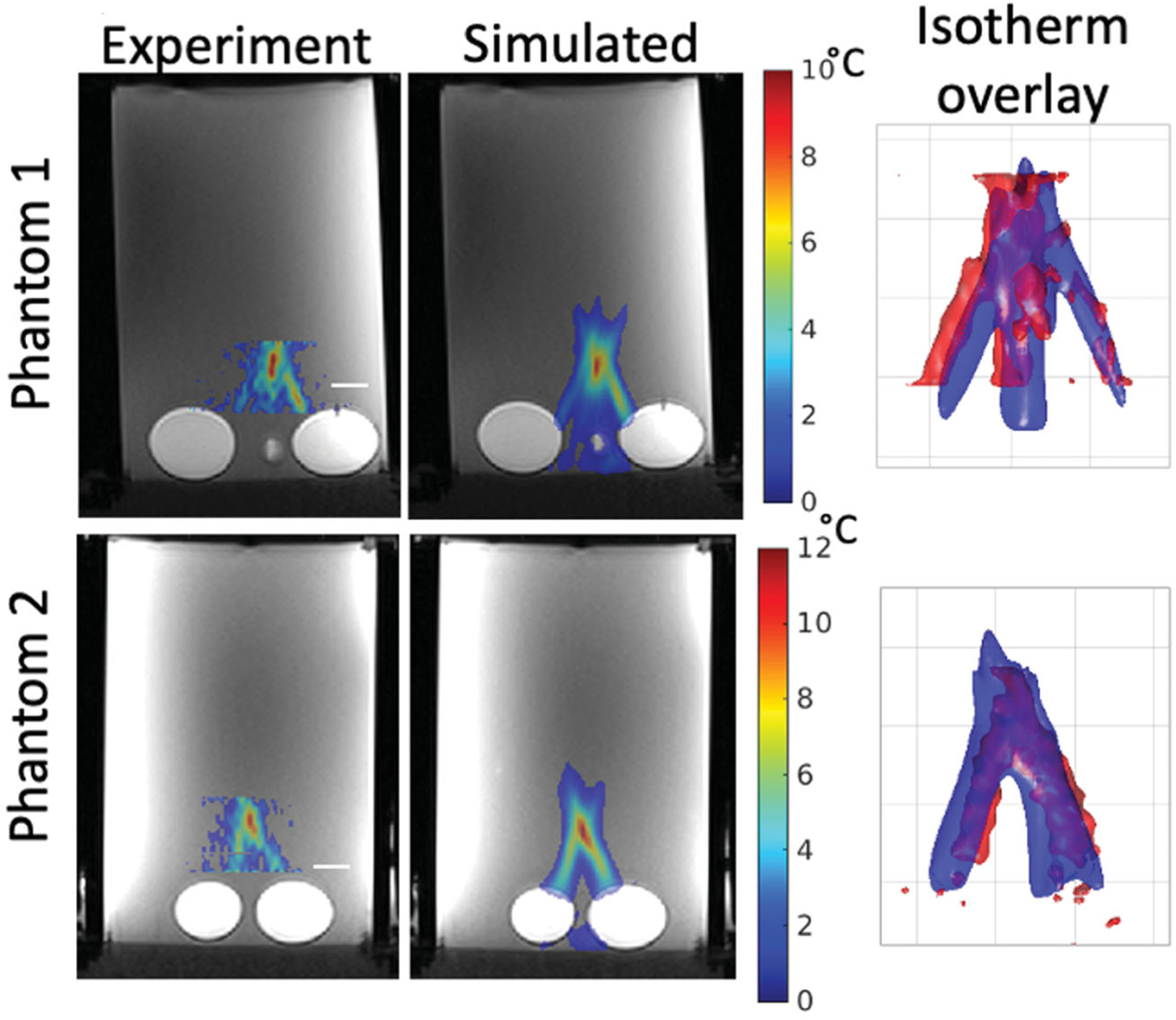
Experimental and simulated temperatures overlaid on an axial T1w MRI image for Sonication 1 of both T-Type Phantoms. Phantom 1 results are shown in the top row and Phantom 2 in the bottom row. The slice is located at the point of peak spatial and temporal temperature rise. The overlap between experiment and simulation result is visualized in the right column, where isotherm surfaces of the simulated (blue) and experimental (red) temperatures are overlaid at a threshold temperature rise of 4 °C. In all cases, the transducer is located at the bottom of the image, similar to the orientation seen in Figure 2. The white scale bars in the left column and the grid lines in the right column denote 1 cm.

Table 4 shows calculated comparison metrics for the temperatures in the two heterogeneous T-Type phantoms. For each T-Type phantom, three separate sonications were performed, so the per-sonication values as well as the mean value ± one standard deviation are reported for each phantom. Figure 4 plots the simulated and experimental temporal peak temperature responses as well as a spatial mean centered at the peak temperature point over a 16-mm^3^ cubic volume for each thermal phantom and each individual sonication. Figure 5 shows the simulated and experimental spatial temperature distributions in the plane containing the peak temperature point overlaid on T1-weighted images for the first sonication in both phantoms. The experimental data were acquired in coronal orientation with a limited field of view (20 mm) in the slice direction, which is why the experimental temperature map appears truncated in the vertical direction. The spatial overlap of the experimental and simulated temperature responses is visualized using isotherm surfaces displaying a 4 °C temperature-rise contour.

## 4. Discussion

Quantitative validation of algorithms intended for clinical MRgFUS treatment planning is important for viable MRgFUS development and translation. This paper has evaluated the hybrid angular spectrum modeling algorithm in aberrating conditions using comparisons to both hydrophone pressure measurements and MR temperature measurements. Although the HAS algorithm is based on several key assumptions (linearity, property uniformity over time, steady-state conditions, and paraxial beam propagation), this study demonstrates that this fast, clinically implementable treatment-planning technique can provide predictions of acoustic pressure and temperature response in strongly heterogeneous phantoms within 11.4% and 10.7% (mean between Phantom 1 and Phantom 2), respectively, when compared to hydrophone measurements and experimental MRTI metrics.

The homogeneous P-Type phantom results that were described prior to the heterogeneous phantom results reveals the degree of variability that can be expected due to uncertainties in the modeling inputs for these simpler homogeneous cases. Because the hybrid angular spectrum method simplifies to the proven traditional angular spectrum approach for a homogeneous model, this variability can be attributed mostly to uncertainties in the input parameters, such as the measurement of medium speed of sound and attenuation, transducer acoustic output power, and hydrophone calibration. Here the HAS simulation results matched the experimental values to within 4–6%. For the heterogeneous phantoms, where the beam was strongly aberrated and the HAS algorithm is tested much more severely, the results showed about twice as much variation (9–11% in pressures and 11% in temperature) between simulation and experiment. The closer 5% match of the HAS pressure patterns to those of k-Wave (widely considered to be a highly accurate beam modeling technique [21]) suggests that about half the larger variation found between simulations and experiment in the heterogeneous patterns may be due to model uncertainties and about half to inaccuracies in the HAS simulation method. Considering the variability expected to be found in patient-specific tissue properties and that is inherent in MR temperature mapping, the variation seen in this study may be comparable in degree to the uncertainty in the assumed input parameters in clinical applications, so the rapid speed of a HAS treatment plan could be an over-riding and significant positive factor.

As mentioned, one benefit of the HAS method is its speed of computation. For example, computation times were recorded for 3D pressure simulations of one of the heterogeneous T-Type models in this study (which was trimmed on its edges and end to a size of 267 × 251 × 300 voxels 0.25 mm isotropic to avoid exceeding the memory of the GPU) both for the HAS method on a CPU alone and for the k-Wave approach using its GPU-implemented version. The computer, running Windows 10, utilized an Intel i5-7600K 3.8 GHz CPU with 4 cores and 32 GB memory. The GPU was an NVIDIA GTX 1070 with 1920 cores. The HAS calculation time with MATLAB 2020b (including two reflection passes) using the CPU with no additional parallelization (but multi-threaded FFT implementation) was 16.5 s. The k-Wave calculation time using the GPU was 599.9 s. (The subsequent thermal modeling was done by the FDTD technique with no parallelization with an additional calculation time of approximately 5 min.) Although both beam calculation times are within the acceptable range for clinical treatment planning, a faster simulation time would be desirable if the beam is expected to be targeted to several different tissue locations. The HAS routine is anticipated to be made faster when it is implemented on a GPU (in the planning stages).

Considering the degrees of variability found for the heterogeneous models, the HAS method did an impressive job in predicting the shape of the highly aberrated beam, as seen in Figures 3 and 5. These figures show beam shapes that are noticeably different from those expected at the tight focus of an unperturbed beam and are a result of the distinct differences in speed of sound and attenuation in the various zones of the phantoms. By design, the phantoms were constructed to emphasize contrast between acoustic properties and therefore to challenge the ability of HAS to handle these inhomogeneities in an environment even more aberrating than expected in human tissues. Within the constraints of the experimental procedures, the patterns match quite well. One measure of the similarity between the experimental and simulated patterns in Figure 3 was the Earth Movers Distance metric, shown to be lower (more similar) than for random patterns. (The reason that all the EMD values were small was that the total “mass” of each pattern was normalized to 1.0 to ensure convergence of the EMD optimization routine.)

The accuracy of any simulation will in part depend on the accuracy of the input parameters. Treatment-planning simulations in thermally based focused ultrasound applications require both acoustic and thermal properties for the modeling algorithms. In this work, properties were directly measured from W-Type phantoms constructed from the same materials as the tested subjects. The property data in Table 2 show that the coefficient of variation for these acoustic and thermal properties are all less than 4% except for acoustic attenuation, where the coefficient of variation ranges from 14% to 41% depending on the gelatin type. These values are in line with the anticipated property variability reported in Johnson et al. [26]. In that study, a sensitivity analysis demonstrated that acoustic attenuation was the largest contributor to simulation output uncertainty. In Figure 4 and Table 3, it can be seen that Sonication 1 in both T-Type phantoms has better qualitative and quantitative agreement to the simulated data across all metrics than the two following sonications. It has been shown in past studies that local temperature changes in gelatin can cause alteration of property values over time, affecting the spatial temperature profile. Specifically, acoustic attenuation has been shown to decrease [36] and specific heat capacity increase [37] with elevated temperatures in gelatin. These combined potential effects can alter local heating and may account for the difference in peak temperature seen between sonication 1 and sonications 2 and 3 in both phantoms. Although it is beyond the scope of this study, some studies have investigated incorporating thermally dynamic tissue properties into simulations [38,39].

Adequate spatial resolution of magnetic resonance thermometry is important to accurately characterize the thermal response. In the MRgFUS sonications described herein, MR thermometry was acquired at a spatial resolution of 1.5 × 1.5 × 3 mm. In [40], it was shown that for the an unaberrated ultrasound beam size similar to that used in the current studies, a spatial acquisition resolution of 1 × 1 × 3 mm achieved accurate characterization of the temperature rise. Since this resolution in this work was slightly larger than the 1 × 1 × 3 mm, it can be assumed that the measured temperatures were underestimated by approximately 5–10%. Therefore, it can be reasoned that even with interpolation of the experimental data, the simulated temperature data would be slightly higher than the experimental temperature data, as is seen in Figure 4.

Implementation of any treatment-planning technique requires the generation of a subject-specific model, either done at a pre-planning session or on the treatment day. The phantoms used in this study were segmented with Dixon fat/water reconstructed images. The assumption of spatially uniform thermal and acoustic properties within each segmented material type (gelatin, oil) is reasonable due to the physical nature of these tissue-mimicking media. The segmentation process utilized automatic tools; however, some manual intervention was required because of the signal inhomogeneity across the MR images due to nonuniformly spaced radiofrequency coil placement. Despite this manual intervention and the variability of the segmentation process, the very good qualitative and quantitative agreement shown by the center of thermal mass agreement and the visual pattern comparisons in Figure 5 indicate that this segmentation variability did not substantially affect the accuracy of the simulation methods. Transducer positioning was also based on the transducer position as observed in the MR images. To minimize the effect of user bias in determining the transducer position, four independent observers identified the position, resulting in a standard deviation of 0.61 mm, on the order of the imaging resolution and therefore within an acceptable amount of variability.

This study has several limitations. The validity of the linear approximation in HAS was not examined since the pressure comparisons using the P-Type phantoms were completed at low acoustic power (2.3 W) to avoid damaging the hydrophone. The thermal T-Type phantoms were evaluated at a higher power (50 W) and non-linear effects may have contributed to inaccuracies in that simulation. Also, the heterogeneous phantoms were constructed with only two components: gelatin and canola oil. While these substances were chosen to emulate the properties of breast fibroglandular tissue and breast fat, the construction is still a substantial simplification of human anatomy. However, this formulation did provide a structure to allow the evaluation of the performance of the HAS method in a largely heterogeneous and phase-aberrated model.

## 5. Conclusions

This work utilized homogeneous and unique heterogeneous phantoms to quantitatively evaluate the performance of the hybrid angular spectrum simulation method in the heterogeneous environment for which it was developed. HAS comparisons with experimental values were done both with pressure measurements and MR temperature imaging.

Normalization of the individual patterns was avoided where possible. The variation was larger (10.2% and 11.1%) for the heterogenous phantoms than for the initial homogeneous phantoms, but at least half of that increase could be attributed to uncertainties of material properties rather than inaccuracies in the modeling approach. The degree of variation reported in this study could be acceptable in treatment-planning situations where tissue-property or other uncertainties are of a similar magnitude to this variation and where the speed of the simulation is of prime importance.

## Figures and Tables

**Table 1.** Details of homogeneous and heterogeneous phantoms used in the study.

Phantom type	Uniformity	Purpose	Size (diameter × height, mm)	Number used in study
Witness (W-Type)	Homogeneous	Speed of sound, attenuation and thermal characterization	57 × 70	*N* = 9 total (3 at each milk concentration)
Pressure (P-Type)	Homogeneous	Pressure mapping	102 × 30	*N* = 9 total (3 at each milk concentration)
	Heterogeneous		102 × 30	*N* = 3
Thermal (T-Type)	Heterogeneous	MR temperature measurements during MRgFUS sonications	102 × 150	*N* = 2

**Table 2.** Measured material properties used for the acoustic and thermal simulations, reported as a mean ± one standard deviation.

Material	Speed of sound (m/s)	Attenuation (Np/cm @ 1.0MHz)	Density (kg/m^3^)	Thermal conductivity (W/m/°C)	Specific heat (J/kg/°C)
Gelatin, 30% milk	1552.7 ± 0.8	0.027 ± 0.011	1044 ± 17	–	–
Gelatin, 50% milk	1565.1 ± 1.1	0.034 ± 0.008	1052 ± 25	–	–
Gelatin, 70% milk	1578.5 ± 1.6	0.056 ± 0.008	1093 ± 32	0.534 ± 0.019	3316 ± 122
Canola oil	1462 ± 5.0	0.008 ± 0.003	940 ± 27	0.184*	1913*

Starred properties were not measured, but taken from the literature [29].

**Table 3.** Metrics used to quantify the comparison between the HAS-calculated pressure patterns and those found by hydrophone measurements and k-Wave simulations.

		Peak pressure difference (%)		Average FWHM difference (%) HAS versus hydrophone	RMSDn (%)	
Phantom type	Phantom composition	HAS versus hydrophone	k-Wave versus hydrophone		HAS versus hydrophone	HAS versus k-Wave
Homogeneous P-Type (*N =* 3 each)	Gelatin, 30% milk	4.3 ± 2.7	–	4.6 ± 1.8	5.7 ± 0.3	–
	Gelatin, 50% milk	1.5 ± 1.3	–	4.4 ± 2.4	5.6 ± 0.3	–
	Gelatin, 70% milk	4.8 ± 1.6	–	4.1 ± 2.6	5.5 ± 0.2	–
Heterogeneous P-Type (*N* = 3)	Gelatin, 70% milk + canola oil	9.0 ± 6.1	5.4 ± 4.1	^−^	11.4 ± 5.1	5.0 ± 1.8

Average FWHM is not calculated for the heterogeneous phantoms due to the strongly aberrated beam shape.

**Table 4.** Comparison of quantitative temperature metrics for the T-Type heterogeneous phantoms.

Exposure conditions	RMSD (°C)	RMSDn (%)	Δ COTM (mm)
Phantom 1			
Sonication 1	1.02	7.1	1.52
Sonication 2	1.67	11.7	2.26
Sonication 3	1.69	11.8	2.31
Mean ± 1 SD	1.46 ± 0.38	10.2 ± 2.7	2.03 ± 0.44
Phantom 1			
Sonication 1	1.36	8.9	2.03
Sonication 2	1.81	12.0	2.23
Sonication 3	1.91	12.6	2.24
Mean ± 1 SD	1.69 ± 0.29	11.1 ± 2.0	2.17 ± 0.12

RMSD, normalized RMSD (RMSDn) and distance between the simulated and experimental center of thermal mass (Δ COTM) are displayed for each individual sonication as well as the mean ± one standard deviation values for each phantom.

## References

[1] Maloney E, Hwang JH. Emerging HIFU applications in cancer therapy. Int J Hyperthermia. 2015;31(3):302–309. 25367011 10.3109/02656736.2014.969789

[2] Elias WJ, Lipsman N, Ondo WG, A randomized trial of focused ultrasound thalamotomy for essential tremor. N Engl J Med. 2016;375(8):730–739. 27557301 10.1056/NEJMoa1600159

[3] Gallay MN, Moser D, Rossi F, MRgFUS pallidothalamic tract-otomy for chronic therapy-resistant Parkinson’s disease in 51 consecutive patients: single center experience. Front Surg. 2019;6:76. 31993437 10.3389/fsurg.2019.00076 PMC6971056

[4] Stewart EA, Rabinovici J, Tempany CMC, Clinical outcomes of focused ultrasound surgery for the treatment of uterine fibroids. Fertil Steril. 2006;85(1):22–29. 16412721 10.1016/j.fertnstert.2005.04.072

[5] Ashrafi AN, Nassiri N, Gill IS, Contrast-enhanced transrectal ultrasound in focal therapy for prostate cancer. Curr Urol Rep. 2018;19(10):87. 30155585 10.1007/s11934-018-0836-6 PMC9084632

[6] Ozhinsky E, Salgaonkar VA, Diederich CJ, MR thermometry-guided ultrasound hyperthermia of user-defined regions using the ExAblate prostate ablation array. J Ther Ultrasound. 2018;6:7. 30123506 10.1186/s40349-018-0115-5 PMC6088423

[7] Huisman M, ter Haar G, Napoli A, International consensus on use of focused ultrasound for painful bone metastases: current status and future directions. Int J Hyperthermia. 2015;31(3): 251–259. 25677840 10.3109/02656736.2014.995237

[8] Brenin DR. Focused ultrasound ablation for the treatment of breast cancer. Ann Surg Oncol. 2011;18(11):3088–3094. 21861221 10.1245/s10434-011-2011-x

[9] Payne A, Merrill R, Minalga E, A breast-specific MR guided focused ultrasound platform and treatment protocol: first-inhuman technical evaluation. IEEE Trans Biomed Eng. 2021;68(3): 893–904. 32784128 10.1109/TBME.2020.3016206 PMC7878578

[10] Ghanouni P, Dobrotwir A, Bazzocchi A, Magnetic resonance-guided focused ultrasound treatment of extra-abdominal desmoid tumors: a retrospective multicenter study. Eur Radiol. 2017; 27(2):732–740. 27147222 10.1007/s00330-016-4376-5 PMC5097700

[11] Pulkkinen A, Hynynen K. Computational aspects in high intensity ultrasonic surgery planning. Comput Med Imaging Graph. 2010; 34(1):69–78. 19740625 10.1016/j.compmedimag.2009.08.001

[12] Dillon CR, Borasi G, Payne A. Analytical estimation of ultrasound properties, thermal diffusivity, and perfusion using magnetic resonance-guided focused ultrasound temperature data. Phys Med Biol. 2016;61(2):923–936. 26741344 10.1088/0031-9155/61/2/923 PMC4879616

[13] Rayleigh JWS. The theory of sound. Vol. 1. New York City: Dover; 1945.

[14] Hudson TJ, Looi T, Pichardo S, Simulating thermal effects of MR-guided focused ultrasound in cortical bone and its surrounding tissue. Med Phys. 2018;45(2):506–519. 29193144 10.1002/mp.12704

[15] Kuznetsov VP. Equations of nonlinear acoustics. Sov Phys Acoust. 1971;16:467–470.

[16] Zabolotskaya EA, Khokhlov RV. Quasi-plane waves in the non-linear acoustics of confined beams. Sov Phys Acoust. 1969;15:35–40.

[17] Solovchuk M, Sheu TW, Thiriet M. Simulation of nonlinear Westervelt equation for the investigation of acoustic streaming and nonlinear propagation effects. J Acoust Soc Am. 2013;134(5): 3931–3942. 24180802 10.1121/1.4821201

[18] Taraldsen G A generalized Westervelt equation for nonlinear medical ultrasound. J Acoust Soc Am. 2001;109(4):1329–1333. 11325104 10.1121/1.1344157

[19] Grinenko A, Wilcox PD, Courtney CRP, Acoustic radiation force analysis using finite difference time domain method. J Acoust Soc Am. 2012;131(5):3664–3670. 22559343 10.1121/1.3699204

[20] Ding D A simplified algorithm for the second order sound fields. J Acoust Soc Am. 2000;108(6):2759–2764. 10.1121/1.1635409 14758992

[21] Martin E, Jaros J, Treeby BE. Experimental validation of k-wave: nonlinear wave propagation in layered, absorbing fluid media. IEEE Trans Ultrason Ferroelectr Freq Control. 2020;67(1):81–91. 31535990 10.1109/TUFFC.2019.2941795

[22] Zeng X, McGough RJ. Evaluation of the angular spectrum approach for simulations of near-field pressures. J Acoust Soc Am. 2008;123(1):68–76. 18177139 10.1121/1.2812579 PMC3408224

[23] Vyas U, Christensen D. Ultrasound beam simulations in inhomogeneous tissue geometries using the hybrid angular spectrum method. IEEE Trans Ultrason Ferroelectr Freq Control. 2012;59(6): 1093–1100. 22711405 10.1109/tuffc.2012.2300

[24] Almquist S, Parker DL, Christensen DA. Rapid full-wave phase aberration correction method for transcranial high-intensity focused ultrasound therapies. J Ther Ultrasound. 2016;4:30. 27980784 10.1186/s40349-016-0074-7 PMC5143441

[25] Dillon CR, Farrer A, McLean H, Experimental assessment of phase aberration correction for breast MRgFUS therapy. Int J Hyperthermia. 2017; 34:1–39. 29278946 10.1080/02656736.2017.1422029

[26] Johnson SL, Christensen DA, Dillon CR, Validation of hybrid angular spectrum acoustic and thermal modelling in phantoms. Int J Hyperthermia. 2018;35(1):578–590. 30320518 10.1080/02656736.2018.1513168 PMC6365205

[27] Leung SA, Webb TD, Bitton RR, A rapid beam simulation framework for transcranial focused ultrasound. Sci Rep. 2019;9(1): 7965. 31138821 10.1038/s41598-019-43775-6 PMC6538644

[28] Farrer AI, Odéen H, de Bever J, Characterization and evaluation of tissue-mimicking gelatin phantoms for use with MRgFUS. J Ther Ultrasound. 2015;3:9. 26146557 10.1186/s40349-015-0030-y PMC4490606

[29] Przybylski R Canola oil thermal properties. Available from: https://fr.canolacouncil.org/media/515239/canola_oil_physical_chemical_properties_1.pdf.

[30] Payne A, Merrill R, Minalga E, Design and characterization of a laterally mounted phased-array transducer breast-specific MRgHIFU device with integrated 11-channel receiver array. Med Phys. 2012;39(3):1552–1560. 22380387 10.1118/1.3685576 PMC3306440

[31] Todd N, Vyas U, de Bever J, The effects of spatial sampling choices of MR temperature measurements. Magn Reson Med. 2011;65(2):515–521 20882671 10.1002/mrm.22636 PMC3015010

[32] CIBC, seg3D: Volumetric image segmentation and visualization. Salt Lake City (UT): Scientific Computing and Imaging Institute (SCI); 2016. Available from: http://www.seg3d.org.

[33] Pennes HH. Analysis of tissue and arterial blood temperatures in the resting human forearm. J Appl Physiol. 1948;1(2):93–122. 18887578 10.1152/jappl.1948.1.2.93

[34] Christensen DA. Ultrasonic bioinstrumentation. New York (NY): John Wiley and Sons, Inc.; 1988.

[35] Rubner Y, Tomasi C, Guibas LJ. The earth mover’s distance as a metric for image retrieval. Int J Comput Vision. 2000;40(2):99–121.

[36] Parker NG, Povey MJW. Ultrasonic study of the gelation of gelatin, phase diagram, hysteresis and kinetics. Food Hydrocoll. 2012;26(1):99–107.

[37] Moraes ICF, Carvalho RA, Bittante AMQB, et al. Film forming solutions based on gelatin and poly(vinyl alcohol) blends: thermal and rheological characterizations. J Food Eng. 2009;95(4): 588–596.

[38] Prakash P, Diederich CJ. Considerations for theoretical modelling of thermal ablation with catheter-based ultrasonic sources: implications for treatment planning, monitoring and control. Int J Hyperthermia. 2012;28(1):69–86. 22235787 10.3109/02656736.2011.630337 PMC3366914

[39] Guntur SR, Choi MJ. Influence of temperature-dependent thermal parameters on temperature elevation of tissue exposed to high-intensity focused ultrasound: numerical simulation. Ultrasound Med Biol. 2015;41(3):806–813. 25638316 10.1016/j.ultrasmedbio.2014.10.008

[40] Todd N, Vyas U, de Bever J, The effects of spatial sampling choices on MR temperature measurements. Magn Reson Med. 2011;65(2):515–521. 20882671 10.1002/mrm.22636 PMC3015010

